# Role of *PTPN22* and *CSK* gene polymorphisms as predictors of susceptibility and clinical heterogeneity in patients with Henoch-Schönlein purpura (IgA vasculitis)

**DOI:** 10.1186/s13075-015-0796-x

**Published:** 2015-10-13

**Authors:** Raquel López-Mejías, Fernanda Genre, Sara Remuzgo-Martínez, Belén Sevilla Pérez, Santos Castañeda, Javier Llorca, Norberto Ortego-Centeno, Begoña Ubilla, Verónica Mijares, Trinitario Pina, Vanesa Calvo-Río, Natalia Palmou, José A. Miranda-Filloy, Antonio Navas Parejo, Diego Argila, Javier Sánchez-Pérez, Esteban Rubio, Manuel León Luque, Juan María Blanco-Madrigal, Eva Galíndez-Aguirregoikoa, J. Gonzalo Ocejo-Vinyals, Javier Martín, Ricardo Blanco, Miguel A. González-Gay

**Affiliations:** Epidemiology, Genetics and Atherosclerosis Research Group on Systemic Inflammatory Diseases, Rheumatology Division, Hospital Universitario Marqués de Valdecilla, IDIVAL, Avenida de Valdecilla, s/n, 39008 Santander, Spain; Department of Medicine, Hospital Universitario San Cecilio, Calle Dr. Oloriz, 16, 18012 Granada, Spain; Rheumatology Department, Hospital Universitario La Princesa, IIS-Princesa, Calle Diego de León, 62, 28006 Madrid, Spain; Epidemiology and Computational Biology Department, School of Medicine, University of Cantabria, and CIBER Epidemiología y Salud Pública (CIBERESP), IDIVAL, Avenida Cardenal Herrera Oria, s/n, 39011 Santander, Spain; Division of Rheumatology, Hospital Universitario Lucus Augusti, Calle Dr. Ochoa, s/n, 27004 Lugo, Spain; Nephrology Department, Hospital Universitario San Cecilio, Calle Dr. Oloriz, 16, 18012 Granada, Spain; Dermatology Department, Hospital Universitario La Princesa, IIS-Princesa, Calle Diego de León, 62, 28006 Madrid, Spain; Rheumatology Department, Hospital Universitario Virgen del Rocío, Avenida Manuel Siurot, s/n, 41013 Sevilla, Spain; Rheumatology Department, Hospital Universitario de Basurto, Avenida de Montevideo, 18, 48013 Bilbao, Spain; Immunology Department, Hospital Universitario Marqués de Valdecilla, Avenida de Valdecilla, s/n, 39008 Santander, Spain; Institute of Parasitology and Biomedicine López-Neyra, CSIC, Avenida del Conocimiento, s/n, 18016 Granada, Spain; Cardiovascular Pathophysiology and Genomics Research Unit, School of Physiology, Faculty of Health Sciences, University of the Witwatersrand, 7 York Road, Parktown 2193, Johannesburg, South Africa

## Abstract

**Introduction:**

To determine whether the PTPN22 (protein tyrosine phosphatase nonreceptor 22)/CSK (c-src tyrosine kinase) pathway is implicated in the susceptibility and clinical heterogeneity of Henoch-Schönlein purpura (HSP) in the largest series of Caucasian HSP patients ever assessed for genetic studies.

**Methods:**

A set of 329 Spanish patients diagnosed with HSP fulfilling the American College of Rheumatology and the Michel et al. classification criteria and 515 sex and ethnically matched controls were recruited in this study. Two well-known *CSK* (*CSK* rs34933034 and *CSK* rs1378942) and two functional *PTPN22* (*PTPN22* rs2476601 (R620W) and *PTPN22* rs33996649 (R263Q)) polymorphisms, previously associated with autoimmunity, were genotyped with TaqMan single nucleotide polymorphism (SNP) genotyping assays.

**Results:**

No significant differences in the genotype and allele frequencies between HSP patients and controls were observed when the *CSK* rs34933034, *CSK* rs1378942, *PTPN22* rs2476601 (R620W) and *PTPN22* rs33996649 (R263Q) polymorphisms were analyzed independently. In keeping with this observation, no significant differences were found when we assessed these polymorphisms combined conforming haplotypes. In addition, there were no differences in the allele or genotype frequencies when HSP patients were stratified according the age at disease onset, sex, presence of arthralgia/arthritis, nephritis or gastrointestinal manifestations.

**Conclusions:**

Our results do not support association between *PTPN22/CSK* and HSP.

**Electronic supplementary material:**

The online version of this article (doi:10.1186/s13075-015-0796-x) contains supplementary material, which is available to authorized users.

## Introduction

Henoch-Schönlein purpura (HSP), also called immunoglobulin A (IgA) vasculitis, is a leukocytoclastic vasculitis characterized by IgA-dominant immune deposits involving mainly the skin as well as other tissues [1]. HSP is more common in children but is not exceptional in adults [1]. The main feature of this vasculitis is a palpable purpura involving predominantly the lower extremities. Besides skin involvement, HSP often causes joint pain and gastrointestinal complications [2]. Nevertheless, renal manifestations constitute the most serious complications of HSP [2] and long-term morbidity and mortality in these patients are mainly due to renal involvement. Although the molecular bases underlying the origin of HSP have not been elucidated yet, environmental and socioeconomical factors are suggested to be involved in the disease pathogenesis [3]. Moreover, the relevance of genetic variants in both susceptibility and HSP clinical heterogeneity has been demonstrated [3–5].

Protein tyrosine phosphatases (PTPs) are critical regulators of T cell signal transduction [6]. Among these molecules, the lymphoid-specific phosphatase (Lyp) is a 110-kDa PTP encoded by the gene *PTPN22* (protein tyrosine phosphatase nonreceptor 22). Lyp is expressed in lymphocytes where it physically associates to the SH3 domain of CSK (c-src tyrosine kinase) [6]. Since this binding has been considered as the most powerful inhibitor of T cell activation, the PTPN22/CSK pathway has been proposed as a master regulator of autoimmunity [7] being a molecular pathway shared by several inflammatory pathologies. In accordance with that, some studies have described an association between two well-known genetic variants located in the *CSK* gene (*CSK* rs34933034 and *CSK* rs1378942) and several immune-mediated disorders such as systemic lupus erythematosus and systemic sclerosis in Caucasian patients [8, 9]. Additionally, a relationship between two functional genetic variants located in the *PTPN22* gene (*PTPN22* rs2476601 (R620W) and *PTPN22* rs33996649 (R263Q)) and some inflammatory diseases (such as type I diabetes, systemic lupus erythematosus and rheumatoid arthritis) has been demonstrated in Caucasians [6, 10, 11]. In line with that, although the genetic influence of *PTPN22/CSK* in several vasculitides (such as giant cell arteritis, Behçet’s disease and antineutrophil cytoplasmic antibody (ANCA)-associated vasculitides [12–14]) has been demonstrated, there is scarce information on the role of *PTPN22/CSK* in HSP. In this regard, only a small cohort of Caucasian patients was assessed to establish the potential implication of the *PTPN22* rs2476601 (R620W) genetic variant in HSP [15].

Taking these considerations together, this study aimed at investigating whether the *PTPN22/CSK* pathway is actually involved in both HSP susceptibility and clinical heterogeneity. For this purpose, we analyzed two well-known *CSK* (*CSK* rs34933034 and *CSK* rs1378942) and two functional *PTPN22* (*PTPN22* rs2476601 (R620W) and *PTPN22* rs33996649 (R263Q)) polymorphisms, previously associated with autoimmunity, in the largest series of Caucasian patients with this vasculitis ever assessed for genetic studies.

## Methods

### Patients and study protocol

A set of 329 Spanish patients with cutaneous vasculitis who fulfilled Michel et al*.* [16] classification criteria for HSP were included in the present study. According to them, they were classified as having HSP if they fulfilled three or more of the following characteristics: palpable purpura, bowel angina, gastrointestinal bleeding, macroscopic or microscopic hematuria, age at disease onset ≤20 years, and no previous history of medications prior to the onset of the disease. Typically, HSP is more common in children. Although less common, HSP may also occur in adults (in individuals older than 20 years). Therefore, the development of this vasculitis in an adult was not an exclusion criterion for the diagnosis of HSP. However, adults presenting with palpable purpura required the presence of other manifestations (other criteria proposed by Michel et al*.*) and, of course, the exclusion of other vasculitis such as ANCA-associated vasculitis was required in these cases. Also, all patients included in this series were required to fulfill the American College of Rheumatology classification criteria for HSP [17].

This collaborative study started 5 years ago. Because of that, the median duration of follow-up of the patients was 1.5 years. Nevertheless, some patients who had suffered this vasculitis before and who still attended outpatients clinics were retrospectively recruited.

Blood samples were obtained from patients recruited from Hospital Universitario Lucus Augusti (Lugo), Hospital Universitario Marqués de Valdecilla (Santander), Hospital Universitario La Princesa (Madrid), Hospital Universitario San Cecilio (Granada), Hospital Universitario Virgen del Rocío (Sevilla) and Hospital Universitario de Basurto (Bilbao). Information on the main clinical features of the whole series of 329 HSP Spanish patients recruited in this study is shown in Table 1. Joint manifestations were defined if arthralgia or peripheral arthritis was observed on examination. For gastrointestinal (GI) manifestations, bowel angina was considered present if there was diffuse abdominal pain that worsened after meals or bowel ischemia usually with bloody diarrhea. GI bleeding was defined as the presence of melena, hematochezia, or a positive test for occult blood in the stool. Renal manifestations were defined to be present if at least one of the following findings was observed: hematuria (≥5 red blood cells/hpf), proteinuria (>300 mg/24 h), nephrotic syndrome (1 g/day/m^2^ body surface area or >3.5 g/day proteinuria with plasma albumin <25 g/l, with or without edema) and nephritic syndrome (i.e., hematuria with at least two of the following: hypertension, raised plasma urea or creatinine, and oliguria). Renal and severe GI manifestations were frequently observed in 34.6 % and 52.9 % of these patients.

**Table 1:** Main clinical features of a series of 329 Spanish patients with HSP

Main characteristics	% (n/N)
Children (age ≤20 years)/adults (age >20 years)	267/62
Male/female	168/161
Age at the onset of the disease (years, median [IQR])	7 [5–18]
Duration of follow-up (years, median [IQR])	1.5 [1–4]
Palpable purpura and/or maculopapular rash	100 (329/329)
Joint manifestations (arthralgia and/or arthritis)	55.6 (183/329)
Gastrointestinal manifestations (if “a” and/or “b”)	52.9 (174/329)
a) Bowel angina	51.4 (169/329)
b) Gastrointestinal bleeding	15.5 (51/329)
Renal manifestations (if any of the following characteristics)	34.6 (114/329)
a) Hematuria	33.7 (111/329)
b) Proteinuria	32.2 (106/329)
c) Nephrotic syndrome	3.0 (10/329)
d) Nephritic syndrome	1.2 (4/329)

*HSP* Henoch-Schönlein purpura, *IQR* interquartile range

A set of 515, sex and ethnically matched controls (median [interquartile range (IQR)]: 48 [27–62] years; 51 % males and 49 % females) without history of cutaneous vasculitis or any other autoimmune disease constituted by blood donors from National DNA Bank Repository (Salamanca, Spain), was also included in the study.

A subject’s written consent was obtained according to the declaration of Helsinki, and the study was approved by the Ethics Committees of Galicia for Hospital Universitario Lucus Augusti, of Cantabria for Hospital Universitario Marqués de Valdecilla, of Madrid for Hospital Universitario La Princesa, of Andalucía for Hospital Universitario San Cecilio and Hospital Universitario Virgen del Rocío, and of País Vasco for Hospital Universitario de Basurto.

### Genotyping

Genomic deoxyribonucleic acid (DNA) was extracted from peripheral blood mononuclear cells using standard methods. The selection of the single nucleotide polymorphisms (SNPs) was based on their previous association with several inflammatory diseases and some types of vasculitis. In this regard, two well-known SNPs located within *CSK*, *CSK* rs34933034 (C__60143137_10) and *CSK* rs1378942 (C___1642446_10), and two functional SNPs located within *PTPN22*, *PTPN22* rs2476601 (R620W) (C__16021387_20) and *PTPN22* rs33996649 (R263Q) (C__25937239_30), were genotyped using TaqMan SNP genotyping assays in a 7900 HT real-time polymerase chain reaction (PCR) system, according to the conditions recommended by the manufacturer (Applied Biosystems, Foster City, CA, USA). PCR was carried out in a total reaction volume of 4 μl with the following amplification protocol: denaturation at 95 °C for 10 min, followed by 45 cycles of denaturation at 92 °C for 15 s and then annealing and extension at 60 °C for 1 min. Negative controls and duplicate samples were included to check the accuracy of genotyping.

### Statistical analysis

All genotype data were checked for deviation from Hardy-Weinberg equilibrium (HWE) using http://ihg.gsf.de/cgi-bin/hw/hwa1.pl [18]. Statistical power for the study was calculated using CaTS – the power calculator for two-stage association studies (http://www.sph.umich.edu/csg/abecasis/CaTS/) [19].

First, comparisons were performed considering all SNPs independently. Both allelic and genotypic frequencies were calculated and compared by *χ*^2^ or Fisher tests when necessary (expected values below 5). Strength of association was estimated using odds ratios (OR) and 95 % confidence intervals (CI). Allelic ORs for *CSK* and *PTPN22* polymorphisms were estimated using the major allele as a reference. Genotype ORs for *CSK* polymorphisms were estimated in three models: (1) using the GG genotype as reference for *CSK* rs34933034, which was compared with each other genotype (i.e.: GA vs*.* GG and AA vs*.* GG) and the AA genotype as reference for *CSK* rs1378942, which was compared with each other genotype (i.e.: AC vs*.* AA and CC vs. AA); (2) the dominant model: AA + GA vs*.* GG (reference) for *CSK* rs34933034 and CC + AC vs*.* AA (reference) for *CSK* rs1378942; (3) the recessive model: AA vs*.* GG + GA (reference) for *CSK* rs34933034 and CC vs*.* AA + AC (reference) for *CSK* rs1378942.

Subsequently, allelic combinations (haplotypes) of both *CSK* and *PTPN22* polymorphisms were performed in order to uncover hidden signals in the *PTPN22/CSK* pathway. Haplotypes were constructed using Haploview v4.2 software; haplotypic frequencies were compared by *χ*2 test and the strength of association was estimated by OR (using the major haplotype as a reference) and 95 % CI.

All analyses were performed with STATA statistical software 12/SE (Stata Corp., College Station, TX, USA).

## Results

### Differences in genotype and allele frequencies between HSP patients and controls

The *CSK* rs34933034, *CSK* rs1378942, *PTPN22* rs2476601 (R620W) and *PTPN22* rs33996649 (R263Q) genotypes distribution were in Hardy-Weinberg equilibrium (Table 2) and for these four SNPs the genotyping success in HSP patients and controls was greater than 98 %. Accordingly, less than 2 % of the samples failed the genotyping process and were removed from our study. This study had >80 % of power to detect genotypic OR >1.4 for *CSK* rs34933034, *CSK* rs1378942, *PTPN22* rs2476601 (R620W) and *PTPN22* rs33996649 (R263Q).

**Table 2:** Genotype and allele frequencies of *CSK* and *PTPN22* gene polymorphisms in HSP patients and controls

		Change			Genotype, n (%)			Allele test	
SNP	Locus	1/2	Sample set	N	1/1	1/2	2/2	*p*	OR [95 % CI]^a^
rs34933034	*CSK*	G/A	HSP patients	329	219 (66.6)	96 (29.2)	14 (4.3)	0.96	1.00 [0.77-1.28]
			Controls^b^	515	337 (65.4)	161 (31.3)	17 (3.3)		
rs1378942	*CSK*	A/C	HSP patients	329	111 (33.7)	170 (51.7)	48 (14.6)	0.22	1.13 [0.93-1.38]
			Controls^c^	515	193 (37.5)	258 (50.1)	64 (12.4)		
rs2476601	*PTPN22*	G/A	HSP patients	329	285 (86.6)	42 (12.8)	2 (0.6)	0.16	1.34 [0.89-1.99]
			Controls^d^	515	462 (89.7)	51 (9.9)	2 (0.4)		
rs33996649	*PTPN22*	C/T	HSP patients	329	314 (95.4)	15 (4.6)	0	0.15	0.67 [0.36-1.22]
			Controls^e^	515	480 (93.2)	34 (6.6)	1 (0.2)		

*HSP* Henoch-Schönlein purpura, *SNP* single nucleotide polymorphism, *N* number of patients, *OR [95 % CI]* odds ratio with 95 % confidence interval

^a^For the minor allele

^b^pHardy-Weinberg equilibrium = 0.67

^c^pHardy-Weinberg equilibrium = 0.12

^d^pHardy-Weinberg equilibrium = 0.64

^e^pHardy-Weinberg equilibrium = 0.63

Table 2 describes the distribution of *CSK* rs34933034, *CSK* rs1378942, *PTPN22* rs2476601 (R620W) and *PTPN22* rs33996649 (R263Q) (considering these genetic variants independently) in HSP patients and controls. As shown in Table 2, no significant differences in the genotype and allele frequencies of these four SNPs between HSP patients and controls were observed (*p* >0.10 in all the cases). Additionally, no statistically significant differences were found when we analyzed *CSK* polymorphisms under different genetic models such as codominant, dominant and recessive (Table S1 in Additional file 1). However, due the relatively low number of *PTPN22* mutant homozygous, these genetic models could not be calculated for *PTPN22* polymorphisms.

In a further step, we assessed *CSK* rs34933034 and *CSK* rs1378942 combined conforming haplotypes. However, the haplotype analysis did not yield additional information, as no differences between HSP patients and controls were found (Table S2 in Additional file 2). It was also the case when the analysis of *PTPN22* rs2476601 (R620W) and *PTPN22* rs33996649 (R263Q) combined conforming haplotypes was performed (Table S2 in Additional file 2).

### Differences in genotype and allele frequencies between HSP patients according to the age at disease onset

Since HSP is generally a benign and self-limited pathology in children and a more severe condition in adults [2], in our study we also assessed if potential differences in *CSK* rs34933034, *CSK* rs1378942, *PTPN22* rs2476601 (R620W) and *PTPN22* rs33996649 (R263Q) could exist in HSP patients stratified according to the age at disease onset. However, as shown in Table 3A and B, no differences in genotype and allele frequencies were detected between HSP in children (age ≤20 years) and adults (age >20 years).

**Table 3:** Genotype and allele frequencies of *CSK* and *PTPN22* gene polymorphisms in HSP patients according to the age at disease onset and the presence/absence of renal and GI manifestations

A. *CSK* polymorphisms												
SNP	Children (Age ≤20 years)				HSP with renal manifestations^a^				HSP with GI manifestations^b^			
	Yes (n = 267)	No (n = 62)	*p*	OR [95 % CI]	Yes (n = 114)	No (n = 215)	*p*	OR [95 % CI]	Yes (n = 174)	No (n = 155)	*p*	OR [95 % CI]
*CSK* rs34933034												
Genotypes												
GG	172 (64.4)	39 (62.9)	-	Ref.	73 (64.0)	138 (64.2)	-	Ref.	122 (70.1)	97 (62.6)	-	Ref.
GA	83 (31.1)	21 (33.9)	0.72	0.90 [0.48-1.71]	37 (32.4)	65 (30.2)	0.77	1.08 [0.64-1.81]	47 (27.0)	49 (31.6)	0.27	0.76 [0.46-1.27]
AA	12 (4.5)	2 (3.2)	0.69	1.36 [0.28-12.9]	4 (3.5)	12 (5.6)	0.43	0.63 [0.14-2.18)	5 (2.9)	9 (5.8)	0.15	0.44 [0.11-1.53]
Alleles												
G	427 (80.0)	99 (79.8)	-	Ref.	183 (80.2)	341 (79.3)	-	Ref.	291 (83.6)	243 (78.4)	-	Ref.
A	107 (20.0)	25 (20.2)	0.98	0.99 [0.60-1.69]	45 (19.7)	89 (20.7)	0.77	0.94 [0.62-1.43]	57 (16.4)	67 (21.6)	0.09	0.71 [0.47-1.07]
*CSK* rs1378942												
Genotypes												
AA	95 (35.6)	18 (29.0)	-	Ref.	44 (38.6)	69 (32.1)	-	Ref.	64 (36.8)	47 (30.3)	-	Ref.
AC	138 (51.7)	32 (51.6)	0.53	0.82 [0.41-1.60]	58 (50.9)	112 (52.1)	0.41	0.81 [0.48-1.37]	89 (51.1)	81 (52.3)	0.38	0.81 [0.48-1.34]
CC	34 (12.7)	12 (19.4)	0.14	0.54 [0.22-1.36]	12 (10.5)	34 (15.8)	0.12	0.55 [0.24-1.24]	21 (12.1)	27 (17.4)	0.11	0.57 [0.27-1.20]
Alleles												
A	328 (61.4)	68 (54.8)	-	Ref.	146 (64.0)	250 (58.1)	-	Ref.	217 (62.4)	175 (56.5)	-	Ref.
C	206 (38.6)	56 (45.2)	0.18	0.76 [0.50-1.16]	82 (36.0)	180 (41.9)	0.14	0.78 [0.55-1.10]	131 (37.6)	135 (43.5)	0.12	0.78 [0.57-1.08]
B. *PTPN22* polymorphisms												
SNP	Children (Age ≤20 years)				HSP with renal manifestations^a^				HSP with GI manifestations^b^			
	Yes (n = 267)	No (n = 62)	*p*	OR [95 % CI]	Yes (n = 114)	No (n = 215)	*p*	OR [95 % CI]	Yes (n = 174)	No (n = 155)	*p*	OR [95 % CI]
*PTPN22* rs2476601												
Genotypes												
GG	229 (85.8)	56 (90.3)	-	Ref.	99 (86.8)	186 (86.5)	-	Ref.	152 (87.4)	133 (85.8)	-	Ref.
GA	37 (13.9)	5 (8.1)	0.23	1.81 [0.67-6.16]	15 (13.2)	26 (12.1)	0.82	1.08 [0.51-2.24]	22 (12.6)	20 (12.9)	0.91	0.96 [0.48-1.95]
AA	1 (0.4)	1 (1.6)	0.28	0.24 [0.003-19.5]	0	3 (1.4)	-	-	0	2 (1.3)	-	-
Alleles												
G	495 (92.7)	117 (94.4)	-	Ref.	213 (93.4)	398 (92.6)	-	Ref.	326 (93.7)	286 (92.3)	-	Ref.
A	39 (7.3)	7 (5.6)	0.51	1.32 [0.56-3.58]	15 (6.6)	32 (7.4)	0.68	0.88 [0.43-1.71]	22 (6.3)	24 (7.7)	0.48	0.80 [0.42-1.53]
*PTPN22* rs33996649												
Genotypes												
CC	256 (95.9)	58 (93.5)	-	Ref.	108 (94.7)	206 (95.8)	-	Ref.	165 (94.8)	149 (96.1)	-	Ref.
CT	11 (4.1)	4 (6.5)	0.43	0.62 [0.18-2.78]	6 (5.3)	9 (4.2)	0.66	1.27 [0.36-4.12]	9 (5.2)	6 (3.9)	0.57	1.35 [0.42-4.74]
TT	0	0	-	-	0	0	-	-	0	0	-	-
Alleles												
C	523 (97.9)	120 (96.8)	-	Ref.	222 (97.4)	421 (97.9)	-	Ref.	339 (97.4)	304 (98.1)	-	Ref.
T	11 (2.1)	4 (3.2)	0.43	0.63 [0.18-2.77]	6 (2.6)	9 (2.1)	0.66	1.26 [0.37-4.03]	9 (2.6)	6 (1.9)	0.58	1.35 [0.42-4.65]

*HSP* Henoch-Schönlein purpura, *GI* gastrointestinal, *SNP* single nucleotide polymorphism, *OR* odds ratio, *CI* confidence interval

^a^If any of the following characteristics: hematuria, proteinuria, nephrotic syndrome and/or nephritic syndrome

^b^If bowel angina and/or gastrointestinal bleeding

### Differences in genotype and allele frequencies between HSP patients according to the presence of nephritis and gastrointestinal manifestations

The allele and genotype frequencies were also examined in HSP patients stratified by the presence of nephritis. Nevertheless, no statistically significant differences between HSP patients with or without renal manifestations were observed (Table 3A and B). This was also the case when HSP patients with severe GI complications (GI bleeding or bowel angina) were compared with those without these complications (Table 3A and B).

### Differences in genotype and allele frequencies between HSP patients according to sex and the presence of joint manifestations (arthralgia or arthritis)

Finally, we assessed if potential differences in genotype and allele frequencies could exist in HSP patients stratified according to sex and the presence of arthralgia or arthritis. However, no statistically significant differences in the genotype and allele frequencies were found between HSP patients stratified according to sex (Table S3 in Additional file 3). It was also the case when HSP patients were stratified according to the presence or absence of joint manifestations (Table S3 in Additional file 3).

## Discussion

Accumulating evidences clearly suggest that a common genetic component may underlie different autoimmune diseases. PTPN22/CSK pathway has been postulated as a potential common genetic factor shared for different autoimmune disorders [6, 8–11].

The vasculitides constitute a heterogeneous group of diseases that often have overlapping clinical and pathological manifestations [20]. Although their complex etiology is far from being completely understood, genetic factors appear to influence the development and progression of these conditions [3–5]. In this regard, an implication of *PTPN22/CSK* in the susceptibility to giant cell arteritis, Behçet’s disease and ANCA-associated vasculitides has been described in Caucasians [12–14]. However, the potential influence of *PTPN22/CSK* in HSP remains unclear. With respect to this, a few years ago Orozco et al*.* did not observe a significant association between the *PTPN22* gene polymorphism and HSP [15]*.* This study was based on the analysis of a single functional polymorphism (*PTPN22* rs2476601 (R620W)) and it was performed in a small cohort of only 57 HSP patients [15]. Accordingly, a potential false negative result could not be excluded due to the underpowered sample size of the study. Because of that, in an attempt to establish if *PTPN22/CSK* is actually involved in HSP, we analyzed two well-known *CSK* and two functional *PTPN22* polymorphisms, previously associated with autoimmunity, in the largest series of Caucasian patients with this vasculitis ever assessed for genetic studies. Our results do not show an implication of *PTPN22/CSK* polymorphisms in the susceptibility to HSP.

The PTPN22/CSK pathway acts as an inhibitor of T cell signaling [6]. T cells appear to be more relevant than B cells in some autoimmune diseases whereas in others it seems to be the opposite. With respect to this, the relevance of IgA-dominant immune deposits in HSP development suggests that the implication of T cells in HSP may be less important than in other autoimmune diseases or other types of vasculitis such as, for example, in giant cell arteritis [1]. In this regard, the lack of association between *PTPN22/CSK* and susceptibility and clinical spectrum of HSP found in our study supports the hypothesis that B cells are more important than T cells in HSP.

In keeping with our findings, no association with *PTPN22/CSK* was also described in other immune-mediated diseases such as multiple sclerosis and ankylosing spondylitis [21, 22]. Taken together, the results in terms of *PTPN22/CSK* association with autoimmune diseases support the notion that different pathogenic mechanisms are involved in the development of polygenic disorders.

The results derived from the present study may be of potential clinical interest. In this respect, since small molecule inhibitors of Lyp would have preventative and/or therapeutic efficacy in patients with a wide range of autoimmune diseases (especially in individuals who are carriers of the *PTPN22* rs2476601 (R620W) genetic variant), the lack of association between the *PTPN22/CSK* polymorphisms pathway and HSP indicate that small molecule inhibitors of Lyp may not have a beneficial effect in patients with this vasculitis.

To conclude, although our cohort of HSP patients constitutes the largest series of Caucasian patients with this vasculitis ever assessed for genetic studies, further large collaborative studies in other populations are needed to fully establish the role of T cell activation polymorphisms in HSP.

## Conclusions

Our results do not support an association between *PTPN22/CSK* gene polymorphisms and HSP. Consequently, small molecule inhibitors of Lyp may not have a beneficial effect in patients with HSP.

## Additional files

Additional file 1: Table S1. Different genetic models of inheritance for *CSK* polymorphisms. (DOC 28 kb) Additional file 2: Table S2.
*CSK* and *PTPN22* haplotype analysis. (DOC 33 kb) Additional file 3: Table S3. Genotype and allele frequencies of *CSK* and *PTPN22* gene polymorphisms in HSP patients according to sex and the presence of joint manifestations (arthralgia or arthritis). (DOC 67 kb)

## Abbreviations

ANCAs: antineutrophil cytoplasmic antibodies
CI: confidence interval
CSK: c-src tyrosine kinase
DNA: deoxyribonucleic acid
GI: gastrointestinal
HSP: Henoch-Schönlein purpura
HWE: Hardy-Weinberg equilibrium
IgA: immunoglobulin-A
IQR: interquartile range
Lyp: lymphoid-specific phosphatase
OR: odds ratio
PCR: polymerase chain reaction
PTPN22: protein tyrosine phosphatase nonreceptor 22
PTPs: protein tyrosine phosphatases
SNPs: single nucleotide polymorphisms
