# Supplementary material for: Role of PTPN22 and CSK gene polymorphisms as predictors of susceptibility and clinical heterogeneity in patients with Henoch-Schönlein purpura (IgA vasculitis)
Source: Arthritis Res Ther. 2015 Oct 13;17:286. doi: 10.1186/s13075-015-0796-x (PMC4603645; doi:10.1186/s13075-015-0796-x)
Supplement: Additional file 1: Table S1. — Different genetic models of inheritance for CSK polymorphisms. (DOC 28 kb) [file 13075_2015_796_MOESM1_ESM.doc]

**Supplementary table 1**: Different genetic models of inheritance for *CSK* polymorphisms.

|  | ***CSK* rs34933034** | | | ***CSK* rs1378942** | | |
| --- | --- | --- | --- | --- | --- | --- |
| **Model** | **Genotypes compared** | **p** | **OR [95% CI]** | **Genotypes compared** | **p** | **OR [95% CI]** |
| Co-dominant | GA/GG (Ref.) | 0.58 | 0.92 [0.67-1.24] | AC/AA (Ref.) | 0.38 | 1.14 [0.84-1.55] |
|  | AA/GG (Ref.) | 0.52 | 1.27 [0.61-2.62] | CC/AA (Ref.) | 0.24 | 1.30 [0.84-2.03] |
| Dominant | AA+GA/GG (Ref.) | 0.73 | 0.95 [0.70-1.28] | CC+AC/AA (Ref.) | 0.27 | 1.18 [0.87-1.59] |
| Recessive | AA/GG+GA (Ref.) | 0.47 | 1.30 [0.58-2.85] | CC/AA+AC (Ref.) | 0.36 | 1.20 [0.78-1.83] |

OR: odds ratio; CI: confidence interval.
