# Supplementary material for: Role of PTPN22 and CSK gene polymorphisms as predictors of susceptibility and clinical heterogeneity in patients with Henoch-Schönlein purpura (IgA vasculitis)
Source: Arthritis Res Ther. 2015 Oct 13;17:286. doi: 10.1186/s13075-015-0796-x (PMC4603645; doi:10.1186/s13075-015-0796-x)
Supplement: Additional file 2: Table S2. — CSK and PTPN22 haplotype analysis. (DOC 33 kb) [file 13075_2015_796_MOESM2_ESM.doc]

**Supplementary table 2.** *CSK* and *PTPN22* haplotype analysis.

| *CSK* Haplotypes | | p | OR [95% CI] |
| --- | --- | --- | --- |
| rs34933034 | rs1378942 |  |  |
| G | A | - | Ref. |
| G | C | 0.11 | 0.82 [0.64-1.05] |
| A | C | 0.24 | 0.85 [0.65-1.12] |
| A | A | 0.27 | 0.32 [0.007-2.89] |
|  |  |  |  |
| *PTPN22* Haplotypes | |  |  |
| rs2476601 | rs33996649 |  |  |
| G | C | - | Ref. |
| A | C | 0.17 | 1.33 [0.86-2.05] |
| G | T | 0.14 | 0.62 [0.29-1.22] |
| A | T | 0.96 | 1.05 [0.08-9.17] |
|  |  |  |  |
| OR: Odds Ratio; CI: confidence Interval. | | | |
