# Supplementary material for: Role of PTPN22 and CSK gene polymorphisms as predictors of susceptibility and clinical heterogeneity in patients with Henoch-Schönlein purpura (IgA vasculitis)
Source: Arthritis Res Ther. 2015 Oct 13;17:286. doi: 10.1186/s13075-015-0796-x (PMC4603645; doi:10.1186/s13075-015-0796-x)
Supplement: Additional file 3: Table S3. — Genotype and allele frequencies of CSK and PTPN22 gene polymorphisms in HSP patients according to sex and the presence of joint manifestations (arthralgia or arthritis). (DOC 67 kb) [file 13075_2015_796_MOESM3_ESM.doc]

**Supplementary table 3**. Genotype and allele frequencies of *CSK* and *PTPN22* gene polymorphisms in HSP patients according to sex and the presence of joint manifestations (arthralgia or arthritis).

| **SNP** | **Sex** | | | | **HSP with arthralgia or arthritis** | | | |
| --- | --- | --- | --- | --- | --- | --- | --- | --- |
|  | Males  (n=168) | Females  (n=161) | p | OR [95% CI] | Yes  (n=183) | No  (n=146) | p | OR [95% CI] |
| ***CSK* rs34933034** |  |  |  |  |  |  |  |  |
| Genotypes |  |  |  |  |  |  |  |  |
| GG | 114 (67.9) | 105 (65.2) | - | Ref. | 122 (66.7) | 88 (60.3) | - | Ref. |
| GA | 45 (26.8) | 51 (31.7) | 0.39 | 0.81 [0.48-1.35] | 55 (30.1) | 50 (34.2) | 0.33 | 0.79 [0.48-1.30] |
| AA | 9 (5.4) | 5 (3.1) | 0.37 | 1.65 [0.48-6.49] | 6 (3.3) | 8 (5.5) | 0.26 | 0.54 [0.15-1.85] |
| Alleles |  |  |  |  |  |  |  |  |
| G | 273 (81.3) | 261 (81.1) | - | Ref. | 299 (81.7) | 226 (77.4) | - | Ref. |
| A | 63 (18.8) | 61 (18.9) | 0.94 | 1.01 [0.67-1.52] | 67 (18.3) | 66 (22.6) | 0.17 | 0.77 [0.51-1.14] |
| ***CSK* rs1378942** |  |  |  |  |  |  |  |  |
| Genotypes |  |  |  |  |  |  |  |  |
| AA | 53 (31.5) | 55 (34.2) | - | Ref. | 67 (36.6) | 52 (35.6) | - | Ref. |
| AC | 88 (52.4) | 85 (52.8) | 0.77 | 1.07 [0.64-1.78] | 95 (51.9) | 75 (51.4) | 0.94 | 0.98 [0.59-1.62] |
| CC | 27 (16.1) | 21 (13.0) | 0.41 | 1.33 [0.64-2.80] | 21 (11.5) | 19 (13.0) | 0.67 | 0.86 [0.39-1.88] |
| Alleles |  |  |  |  |  |  |  |  |
| A | 194 (57.7) | 195 (60.6) | - | Ref. | 229 (62.6) | 179 (61.3) | - | Ref. |
| C | 142 (42.3) | 127 (39.4) | 0.46 | 1.12 [0.81-1.55] | 137 (37.4) | 113 (38.7) | 0.74 | 0.95 [0.68-1.32] |
| ***PTPN22* rs2476601** |  |  |  |  |  |  |  |  |
| Genotypes |  |  |  |  |  |  |  |  |
| GG | 148 (88.1) | 134 (83.2) | - | Ref. | 159 (86.9) | 126 (86.3) | - | Ref. |
| GA | 19 (11.3) | 26 (16.1) | 0.20 | 0.66 [0.33-1.31] | 24 (13.1) | 18 (12.3) | 0.87 | 1.05 [0.52-2.16] |
| AA | 1 (0.6) | 1 (0.6) | 0.94 | 0.90 [0.01-71.6] | 0 | 2 (1.4) | - | - |
| Alleles |  |  |  |  |  |  |  |  |
| G | 315 (93.8) | 294 (91.3) | - | Ref. | 342 (93.4) | 270 (92.5) | - | Ref. |
| A | 21 (6.3) | 28 (8.7) | 0.23 | 0.7 [0.37-1.31] | 24 (6.6) | 22 (7.5) | 0.62 | 0.86 [0.45-1.65] |
| ***PTPN22* rs33996649** |  |  |  |  |  |  |  |  |
| Genotypes |  |  |  |  |  |  |  |  |
| CC | 163 (97.0) | 151 (93.8) | - | Ref. | 172 (94.0) | 142 (97.3) | - | Ref. |
| CT | 5 (3.0) | 10 (6.2) | 0.16 | 0.46 [0.12-1.53] | 11 (6.0) | 4 (2.7) | 0.15 | 2.27 [0.65-9.96] |
| TT | 0 | 0 | - | - | 0 | 0 | - | - |
| Alleles |  |  |  |  |  |  |  |  |
| C | 331 (98.5) | 312 (96.9) | - | Ref. | 355 (97.0) | 288 (98.6) | - | Ref. |
| T | 5 (1.5) | 10 (3.1) | 0.16 | 0.47 [0.12-1.53] | 11 (3.0) | 4 (1.4) | 0.16 | 2.23 [0.65-9.69] |
|  |  |  |  |  |  |  |  |  |

HSP: Henoch-Schönlein purpura; SNP: single nucleotide polymorphism; OR: odds ratio; CI: confidence interval.
